# Exploratory NMR-based metabolomics reveals transient lipoprotein changes during prolonged computer gaming

**DOI:** 10.1007/s11306-026-02502-9

**Published:** 2026-07-03

**Authors:** Kasper Bygum Krarup, Henrik Bygum Krarup, Inge Søkilde Pedersen, Morten Mørk, Søren Risom Kristensen, Aase Handberg, Reinhard Wimmer, Hien Thi Thu Nguyen

**Affiliations:** 1 Department of Geriatrics, Aalborg University Hospital, Aalborg, Denmark; 2 Department of Molecular Diagnostics, Aalborg University Hospital, Gistrup, Denmark; 3 Department of Clinical Medicine, Aalborg University, Gistrup, Denmark; 4 Department of Clinical Biochemistry, Regionshospital Nordjylland, Hjørring, Denmark; 5 Department of Clinical Biochemistry, Aalborg University Hospital, Gistrup, Denmark; 6 Department of Chemistry and Bioscience, Aalborg University, Aalborg, Denmark

**Keywords:** LAN party, Long computer gaming, Gaming, Cardiometabolic health, NMR, Metabolomics, Physical inactivity

## Abstract

**Introduction:**

Extended computer gaming is characterized by prolonged sedentary activity, disrupted sleep, emotional stress, and unrestricted intake of energy-dense foods and beverages.

**Objectives:**

This study explored short-term metabolic responses and recovery following prolonged gaming in healthy young men.

**Methods:**

Nine healthy male participants (mean age 25.8 ± 2.6 years) took part in a controlled local area network (LAN) gaming event consisting of two 18-hour gaming sessions separated by a 6-hour sleep period. Serum samples were collected at multiple time points during the intervention and at a 5-day follow-up. Metabolomic profiling was performed using proton nuclear magnetic resonance (^1^H-NMR)-based metabolomics.

**Results:**

Temporal variation in serum metabolomic profiles was observed during prolonged gaming, particularly in lipid-related measures, including very low-density lipoprotein (VLDL) subclasses. Exploratory multivariate analyses showed separation between early and later time points during the intervention. At five days following the gaming sessions, metabolomic profiles showed substantial overlap with baseline. Metabolites that varied during the gaming period generally returned toward baseline concentrations, although inter-individual variability was observed.

**Conclusions:**

Prolonged computer gaming was associated with transient alterations in serum metabolomic profiles in healthy young men, most notably in lipid-related measures. Metabolomic profiles at five days showed convergence toward baseline following a single gaming episode. These findings provide time-resolved insight into metabolomic variation under a real-world behavioral exposure. Further studies are required to determine whether repeated exposure to similar conditions is associated with cumulative metabolic changes over time.

**Supplementary Information:**

The online version contains supplementary material available at https://doi.org/10.1007/s11306-026-02502-9.

## Introduction

Over the past decades, sedentary behaviors have become increasingly prevalent, particularly among adolescents and young adults. Computer gaming represents a common leisure activity within this population and is often accompanied by prolonged sitting, irregular sleep patterns, and frequent consumption of energy-dense foods and beverages (Altintas et al., 2019; Patterson et al., 2018; Strasburger et al., 2012). Although video gaming can elicit modest increases in physiological responses such as heart rate compared with passive screen time (e.g., television), traditional gaming remains within the sedentary metabolic range (≤ 1.5 METs) as defined by the Sedentary Behaviour Research Network (Lanningham-Foster et al., 2006; Tremblay et al., 2017).

Accumulated sedentary time has been associated with adverse cardiometabolic outcomes, including cardiovascular mortality and type 2 diabetes, independent of overall physical activity levels (Hadgraft et al., 2021; Patterson et al., 2018; Wu et al., 2022). Mechanistic and clinical research further suggests that prolonged sitting may influence glucose regulation, lipid metabolism, and vascular function (Franssen et al., 2025; Heinonen, 2024). Systematic reviews have shown that prolonged sedentary time may influence biomarkers relevant to lipid metabolism, although most studies to date have focused on chronic exposures and mixed-age populations (Ylinen et al., 2024).

Experimental studies demonstrate that interrupting sedentary time can acutely modify postprandial metabolic responses, but the frequency and intensity thresholds required to elicit meaningful lipid-related changes remain unclear (Benatti & Ried-Larsen, 2015; Peddie et al., 2013). Together, these findings highlight the need to better understand short-term metabolic responses to concentrated periods of uninterrupted sedentary behavior.

Extended gaming sessions, such as local area network (LAN) events, involve many consecutive hours of computer use, sustained mental engagement, disrupted sleep schedules, and unrestricted access to snacks and beverages. While such sessions are not representative of typical daily behavior, they constitute a real-world gaming scenario that combines multiple behavioral stressors within a short time frame. These conditions provide a unique opportunity to examine acute metabolic responses to prolonged sedentary behavior and behavioral disruption in otherwise healthy individuals.

High-throughput nuclear magnetic resonance (NMR)-based metabolomics enables detailed characterisation of circulating lipoprotein subclasses, lipid fractions, and related biomarkers, providing sensitive insight into short-term metabolic perturbations. With specific relevance to cardiometabolic risk, changes in very low-density lipoprotein (VLDL) particles and triglyceride-rich lipoprotein measures are of particular interest due to their roles in postprandial lipid handling and atherogenic processes (Blake et al., 2002; Krauss, 2010; McGarrah et al., 2023; Packard & Shepherd, 1997; Wang et al., 2017).

The present study aimed to characterize temporal changes in serum metabolomic profiles during prolonged computer gaming under typical LAN party conditions and to assess metabolic recovery in the days following the intervention, focusing on describing acute metabolic responses to a well-defined gaming scenario and the short-term recovery trajectory in healthy young men.

## Materials and methods

### Study participants

Nine healthy young male volunteers were recruited to participate in the study. Participants had a mean age of 25.8 ± 2.6 years and were either full-time students or employed. All participants were experienced gamers who regularly engaged in prolonged gaming sessions and reported gaming approximately 1–5 h daily. Recruitment was conducted through local e-sports clubs, gaming-related online message boards, e-sports instructors, and word of mouth (Kasper B. Krarup et al. 2024a, b; Kasper Bygum Krarup et al. 2024a, b).

Eligibility criteria included healthy male adults aged > 18 years, while exclusion criteria included anemia, hypertension, diabetes mellitus, known metabolic or cardiovascular disease, and regular medication use. Baseline clinical assessment included self-reported medical history, anthropometric measurements, blood pressure, heart rate, and routine biochemical and hematological screening, all of which were within normal reference ranges (Kasper B. Krarup et al. 2024a, b).

The study protocol has been described previously (Kasper Bygum Krarup et al., 2022). Briefly, participants underwent a structured prolonged gaming intervention and were monitored throughout the study period with repeated measurements of body weight, blood pressure, and heart rate. Continuous electrocardiography (ECG) was performed during the intervention.

Prior to the study, participants were instructed to refrain from strenuous physical activity and heavy physical work during the preceding week. They were encouraged to maintain their habitual diet while avoiding excessive intake of energy-dense “junk” foods; however, dietary intake was not standardized or quantitatively recorded before the intervention.

Written informed consent was obtained from all participants before enrolment. The study protocol was approved by The North Denmark Region Committee on Health Research Ethics (approval number N-20180011; EudraCT number 2019–004091).

### Study design

**Fig. 1:**
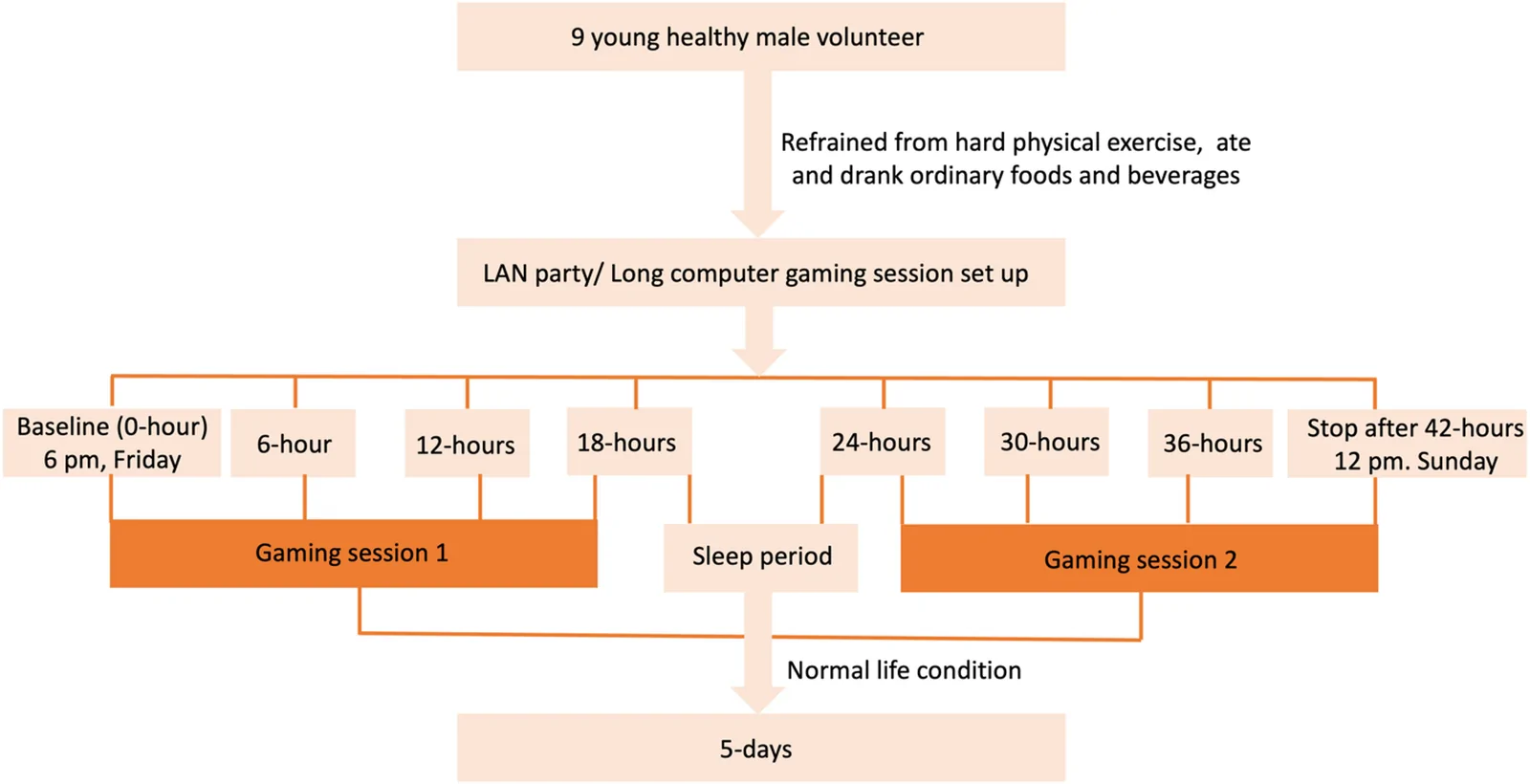
Study flowchart outlining the experimental design

The study was designed to examine metabolic responses to prolonged computer gaming conducted under typical LAN party conditions. Participants took part in two consecutive 18-hour gaming sessions separated by a 6-hour sleep period, resulting in a total intervention duration of 42 h (Fig. 1). Gaming took place in a controlled indoor setting designed to reflect a real-world LAN party environment, with participants seated at individual computer stations throughout the sessions.

Participants were unrestricted regarding game selection and were allowed to play games of their choice individually or competitively. The most frequently played games during the intervention were multiplayer and first-person shooter games.

During the gaming periods, participants had unrestricted access to food and beverages, including chips, candy, cookies, cereal products, pizza, hamburgers, sugar-sweetened beverages, energy drinks, coffee, milk, and chocolate milk. Food and beverage intake was monitored using participant food diaries, and caloric intake data from the intervention have been reported previously (Kasper B. Krarup et al. 2024a, b; Kasper Bygum Krarup et al. 2024a, b).

Between the two gaming sessions, participants rested in designated study-center facilities adjacent to the gaming area and obtained approximately 4–5 h of sleep before resuming gaming. Sleep during this interval was not experimentally controlled or objectively monitored.

### Blood sampling and biochemical measurements

Venous blood samples were collected at baseline (START), at regular 6-hour intervals during the gaming sessions, and again five days after completion of the intervention. All blood samples were obtained with participants in a seated position.

For metabolomics analyses, blood samples were collected by venipuncture and processed immediately after sampling. Samples were centrifuged at 3000 rpm (1875 × g) for 10 min at room temperature, after which serum aliquots were stored at −80 °C until analysis (Kasper Bygum Krarup et al. 2024a, b).

Routine biochemical analyses were performed using accredited clinical laboratory procedures at the Department of Clinical Biochemistry, Aalborg University Hospital. Analyses included measurements obtained on Cobas 8000 Modular Analyzer, Sysmex XN-9000 Hematology Analyzer, and ABL800 FLEX Blood Gas Analyzer platforms, as previously described (Kasper Bygum Krarup et al. 2024a, b).

### NMR metabolomics analysis

Serum metabolomic profiling was performed using a high-throughput proton nuclear magnetic resonance (^1^H-NMR) spectroscopy platform (Nightingale Health, Helsinki, Finland), which enables simultaneous quantification of lipoprotein particle concentrations, lipid subclasses, fatty acid composition, and selected low-molecular-weight metabolites.

The platform quantifies over 200 metabolic measures, including lipid concentrations across 14 lipoprotein subclasses, apolipoproteins, fatty acids, and routine clinical lipid parameters. Detailed technical specifications, analytical performance, and validation of this platform have been described previously (Würtz et al., 2017).

All samples were processed according to standardized protocols provided by the manufacturer. Internal quality control procedures were applied to ensure analytical consistency across batches. No samples were excluded based on quality control criteria.

### Data preprocessing and statistical analysis

All statistical analyses were conducted using R (version 3.5.3) with relevant metabolomics and statistical packages, including MetaboAnalystR, ggsci, ggfortify, and ggplot2.

#### Data preprocessing

Metabolite concentrations were inspected for completeness and distributional properties prior to analysis. Metabolites with greater than 20% missing values across samples were prespecified for exclusion; however, no quantified metabolite exceeded this threshold, and therefore no imputation or variable exclusion was required. Data preprocessing and statistical analyses were performed in R using the packages MetaboAnalystR, ggsci, ggfortify, and ggplot2. Color palettes were generated using the colorRampPalette function in R in combination with palettes from the ggsci package.

Variables exhibiting substantial right-skewness or non-normal distributions, based on visual inspection of histograms and quantile-quantile plots, were log-transformed prior to statistical analysis where appropriate to improve distributional symmetry and satisfy model assumptions. To reduce the influence of scale differences and facilitate comparison across metabolites, data were subsequently autoscaled (mean-centered and divided by the standard deviation) prior to multivariate analyses. For heatmap visualization, metabolite values were additionally expressed as row-wise z-scores, representing the number of standard deviations from the mean concentration for each metabolite across all samples.

Six time points (START, 6 h, 18 h, 24 h, 30 h, and 42 h) were included in multivariate analyses to facilitate consistent visualization of temporal metabolomic patterns across the gaming intervention.

#### Multivariate analysis

Unsupervised principal component analysis (PCA) was performed to explore the overall variance structure of the dataset and identify potential outliers. Hierarchical clustering combined with heatmap visualization was used to examine similarity patterns across samples and time points.

Sparse partial least squares discriminant analysis (sPLS-DA) was applied as an exploratory pattern-recognition approach to visualize temporal metabolomic variation and identify metabolites contributing most strongly to differences between time points (Lê Cao et al., 2011; Puchades-Carrasco et al., 2016). Given the repeated-measures design and limited sample size, the sPLS-DA models were interpreted primarily as descriptive visualization and variable selection rather than as predictive classification models.

Internal 5-fold cross-validation was performed to assess model stability; classification error rates varied across pairwise comparisons and model components. Loading plots were used to identify metabolites contributing most strongly to separation between groups, and variables were ranked according to the absolute magnitude of their loading values.

#### Univariate analysis

To identify metabolites exhibiting significant temporal variation, repeated-measures analysis of variance (ANOVA) was performed across all quantified metabolites to assess differences across time points (START, 6 h, 12 h, 18 h, 24 h, 30 h, 36 h, and 42 h). When the overall ANOVA result was statistically significant, Fisher’s least significant difference (LSD) post hoc test was applied for exploratory pairwise comparisons between time points. For each metabolite, p-values were adjusted for multiple comparisons using the Benjamini–Hochberg false discovery rate (FDR) method. Statistical significance was defined as an adjusted p-value (q-value) < 0.05. Results from both univariate and multivariate analyses were subsequently compared to identify metabolites showing consistent temporal variation across analytical approaches.

#### Software and reproducibility

Multivariate analyses were implemented using a customized implementation of the MetaboAnalystR package (version 4.0). Enhancements to the codebase were incorporated into the official repository. All analyses were performed in a reproducible computational environment, and code is available upon reasonable request.

## Results

### Global metabolomic changes during prolonged gaming

Unsupervised principal component analysis (PCA) was performed to explore the overall structure of the metabolomic data and identify potential outliers. The first principal components captured the major sources of variance across samples, and no clear outliers were identified.

**Fig. 2:**
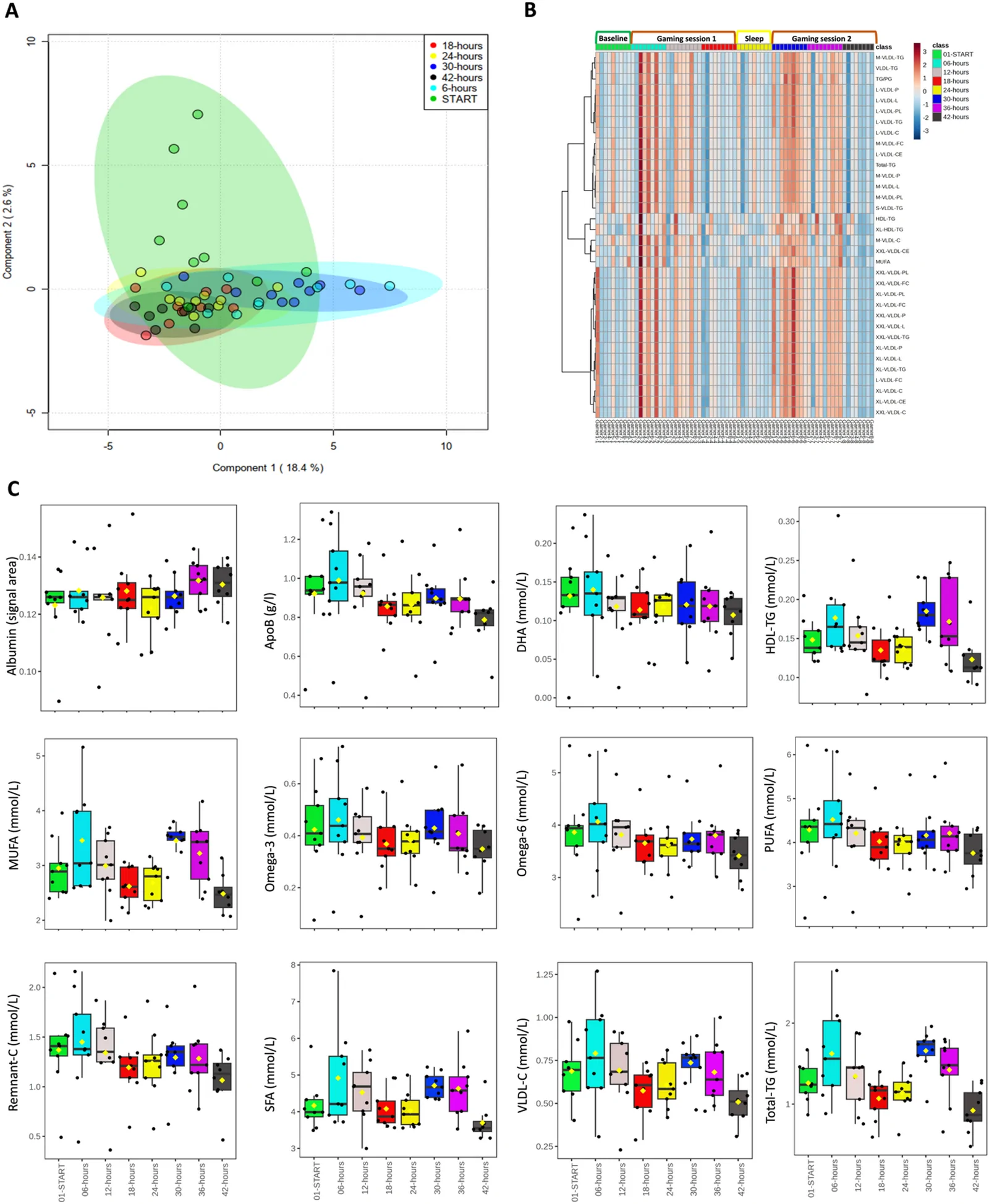
Global metabolomic patterns and health-related metabolites during prolonged gaming. **A** sPLS-DA score plot showing serum metabolomic profiles across six time points: START (green), 6 h (light grey), 18 h (red), 24 h (yellow), 30 h (blue), and 42 h (dark grey). **B** Clustered heatmap of top 34 most variable metabolites across time points: START (green), 6 h (light blue), 12 h (light grey), 18 h (red), 24 h (yellow), 30 h (blue), 36 h (purple), and 42 h (dark grey) Colors represent row-wise standardized values (z-scores), calculated by mean-centering and scaling each metabolite across all samples. Red indicates relative increases and blue indicates relative decreases compared with the metabolite’s overall mean. Values range from − 3 to + 3 standard deviations and are unitless. **C** Biochemical changes in twelve metabolites considered biomarkers of a healthy physiological state: albumin, apolipoprotein B (ApoB), docosahexaenoic acid (DHA), triglycerides in high-density lipoprotein (HDL-TG), monounsaturated fatty acids (MUFA), omega-3 fatty acids (Omega-3), omega-6 fatty acids (Omega-6), polyunsaturated fatty acids (PUFA), remnant cholesterol (Remnant-C), saturated fatty acids (SFA), very low-density lipoprotein cholesterol (VLDL-C), and total triglycerides (Total-TG). In panel C, box plots display the median (horizontal line within the box), interquartile range (box representing the 25th–75th percentiles), and whiskers extending to the most extreme data points within 1.5 × the interquartile range. Individual observations are shown as black dots, and yellow diamonds indicate the mean value at each time point

**Fig. 3:**
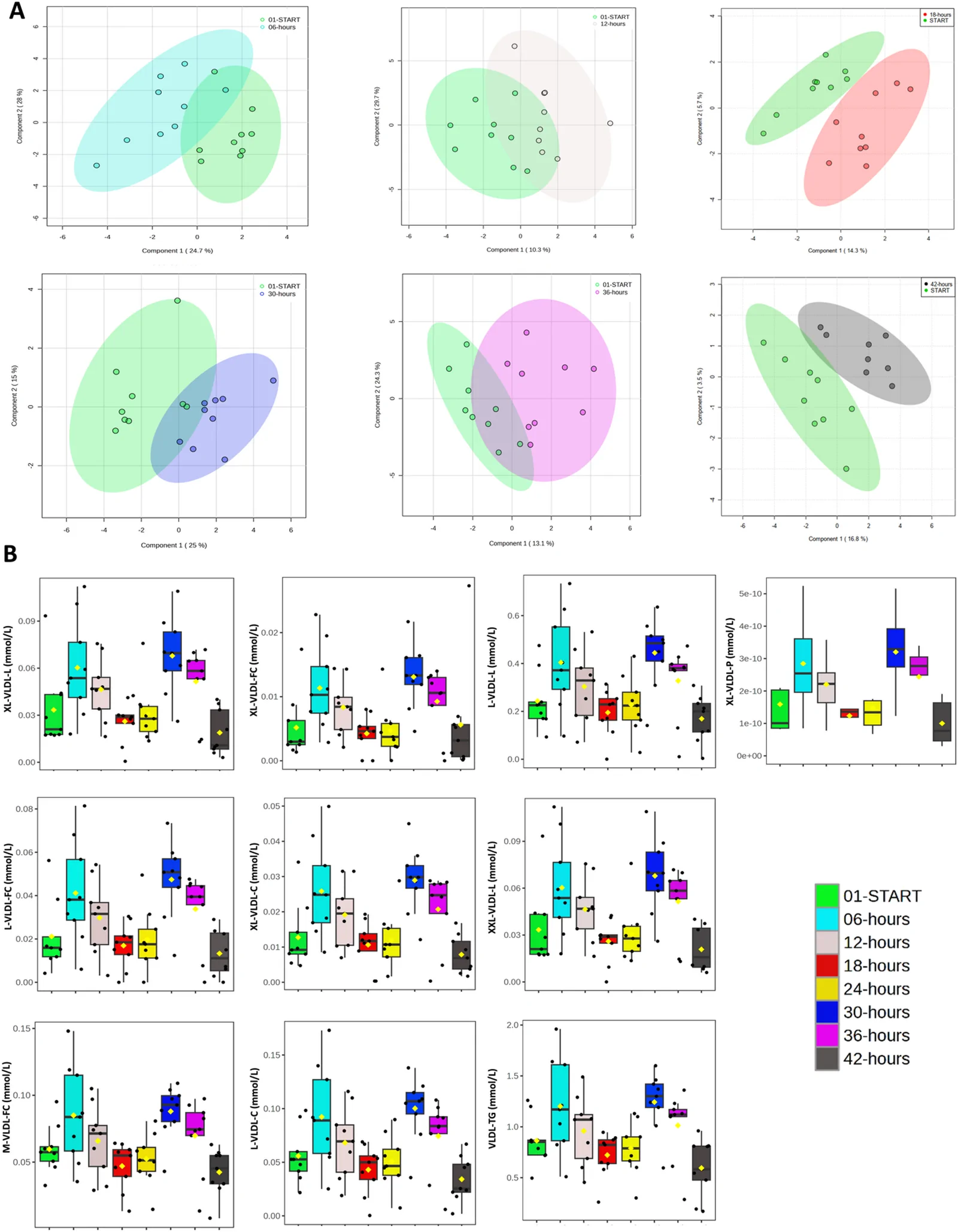
Lipoprotein subclass measures most strongly contributing to temporal differences during prolonged gaming. **A** Pairwise sPLS-DA score plots comparing START versus subsequent time points: START vs. 6 h, START vs. 12 h, START vs. 18 h, START vs. 30 h, START vs. 36 h, and START vs. 42 h. **B** Ten metabolites most strongly contributing to the exploratory separation over the 42 h session: total lipids in very large VLDL (XL-VLDL-L), free cholesterol in XL-VLDL (XL-VLDL-FC), total lipids in large VLDL (L-VLDL-L), particle concentration of XL-VLDL (XL-VLDL-P), free cholesterol in L-VLDL (L-VLDL-FC), cholesterol in XL-VLDL (XL-VLDL-C), total lipids in extremely large VLDL (XXL-VLDL-L), free cholesterol in medium VLDL (M-VLDL-FC), cholesterol in L-VLDL (L-VLDL-C), and triglycerides in VLDL (VLDL-TG). In panel B, box plots display the median (horizontal line within the box), interquartile range (box representing the 25th–75th percentiles), and whiskers extending to the most extreme data points within 1.5 × the interquartile range. Individual observations are shown as black dots, and yellow diamonds indicate the mean value at each time point

Exploratory sparse partial least squares discriminant analysis (sPLS-DA) demonstrated gradual temporal separation of serum metabolomic profiles during the gaming intervention (Fig. 2A). Early time points (START) clustered separately from later time points of each gaming Sect. (18 h, 42 h), whereas intermediate time points showed partial overlap, consistent with progressive temporal variation in metabolomic profiles across the intervention period. The 5-day follow-up samples were analyzed separately to focus Figs. 2 and 3 on acute temporal metabolomic responses during the gaming intervention.

Hierarchical clustering of the 34 most variable metabolic measures further demonstrated time-dependent metabolomic patterns (Fig. 2B). Samples from later time points exhibited similar clustering patterns, whereas baseline samples formed a distinct cluster. Heatmap visualization was based on row-wise standardized values (z-scores), reflecting relative deviations from the mean concentration for each metabolite across samples.

### Temporal changes in selected metabolites

Biochemical changes in twelve metabolites previously proposed as markers of a favorable metabolic profile were examined across time points (Fig. 2C) (Würtz et al., 2017). Albumin concentrations remained relatively stable during the early phase of the intervention and increased toward later time points (36 h and 42 h). Lipid-related measures, including total triglycerides (Total-TG), very low-density lipoprotein cholesterol (VLDL-C), and remnant cholesterol, exhibited temporal variation during the gaming sessions. Similar temporal patterns were observed for fatty acid measures, including monounsaturated fatty acids (MUFA), polyunsaturated fatty acids (PUFA), and omega-3 and omega-6 fatty acids.

Across these metabolites, changes were most apparent at later time points of each gaming section, particularly at 18 h and 42 h, although the magnitudes of variation differed between individuals.

### Lipoprotein subclass responses during prolonged gaming

To further characterize temporal variation across the lipoprotein spectrum, exploratory sPLS-DA models were constructed to compare baseline metabolomic profiles with those obtained at subsequent time points (6 h, 12 h, 18 h, 30 h, 36 h, and 42 h). These analyses were used to visualize temporal metabolomic patterns and identify metabolites contributing most strongly to separation between time points.

The sPLS-DA score plots showed temporal separation between baseline and later time points (Fig. 3A). Early time points (6 h, 12 h) showed partial overlap with baseline samples, whereas later time points within each gaming Sect. (18 h, and 42 h) exhibited greater separation.

Internal 5-fold cross-validation was performed to assess model stability across the exploratory pairwise sPLS-DA models. Error rates varied across pairwise comparisons and model components, with the lowest observed classification error rates ranging from 16.7% to 33.3% depending on the comparison (Supplementary Table S1). Error rates generally increased with additional model components, supporting the use of lower-dimensional models for exploratory visualization and interpretation.

The ten metabolites contributing most strongly to temporal separation across the gaming period are shown in Fig. 3B. These metabolites were predominantly lipids and cholesterol measures within very large and large VLDL subclasses, including total lipids in very large VLDL (XL-VLDL-L), free cholesterol in XL-VLDL (XL-VLDL-FC), total lipids in large VLDL (L-VLDL-L), particle concentration of XL-VLDL (XL-VLDL-P), free cholesterol in large VLDL (L-VLDL-FC), cholesterol in XL-VLDL (XL-VLDL-C), total lipids in extremely large VLDL (XXL-VLDL-L), free cholesterol in medium VLDL (M-VLDL-FC), cholesterol in large VLDL (L-VLDL-C), and triglycerides in VLDL (VLDL-TG). Variables were ranked according to the absolute magnitude of their loading values.

Across lipoprotein subclasses, temporal variation was most apparent in the lipid and cholesterol content of large VLDL particles, while smaller VLDL, LDL, and HDL subclasses showed comparatively less variation over time.

In total, 128 NMR-derived metabolic measures were quantified across seven functional metabolite categories (Supplementary Table S2).

### Univariate analysis of temporal metabolite changes

Repeated-measures analysis identified 34 of 128 NMR-derived metabolic measures with a significant time effect after Benjamini–Hochberg false discovery rate (FDR) correction (q < 0.05). Significant metabolites were predominantly lipid-related measures, particularly very low-density lipoprotein (VLDL) subclass variables and triglyceride-rich lipoprotein measures.

The strongest temporal effects were observed for very large and large VLDL subclass measures, including cholesterol esters in extremely large VLDL (XXL-VLDL-CE), cholesterol in XXL-VLDL (XXL-VLDL-C), cholesterol esters in XL-VLDL (XL-VLDL-CE), cholesterol in XL-VLDL (XL-VLDL-C), free cholesterol in XL-VLDL (XL-VLDL-FC), total lipids in XXL-VLDL (XXL-VLDL-L), and particle concentration of XXL-VLDL (XXL-VLDL-P), all of which demonstrated highly significant temporal variation after FDR correction (Supplementary Table S3).

Additional significant temporal effects were identified for multiple triglyceride-rich lipoprotein measures, including total triglycerides (Total-TG), triglycerides in XL-VLDL (XL-VLDL-TG), triglycerides in large VLDL (L-VLDL-TG), triglycerides in total VLDL (VLDL-TG), triglycerides in medium VLDL (M-VLDL-TG), triglycerides in small VLDL (S-VLDL-TG), triglycerides in extra-large HDL (XL-HDL-TG), and HDL triglycerides (HDL-TG). Significant variation was also observed for several phospholipid and particle concentration measures within large and medium VLDL subclasses, including XL-VLDL-PL, XXL-VLDL-PL, L-VLDL-PL, M-VLDL-PL, L-VLDL-P, and M-VLDL-P. Monounsaturated fatty acids (MUFA) also demonstrated significant temporal variation across the gaming sessions.

The metabolites demonstrating the strongest univariate temporal effects largely overlapped with those contributing most strongly to the exploratory sPLS-DA separation patterns, supporting consistency between the multivariate and univariate analyses.

Full results from the univariate analyses, including F-statistics, nominal p-values, FDR-adjusted q-values for all quantified metabolites, are provided in Supplementary Table S3. Internal cross-validation error rates for the pairwise sPLS-DA models are also provided in Supplementary Table S1.

### Metabolic response following a short sleep period

**Fig. 4:**
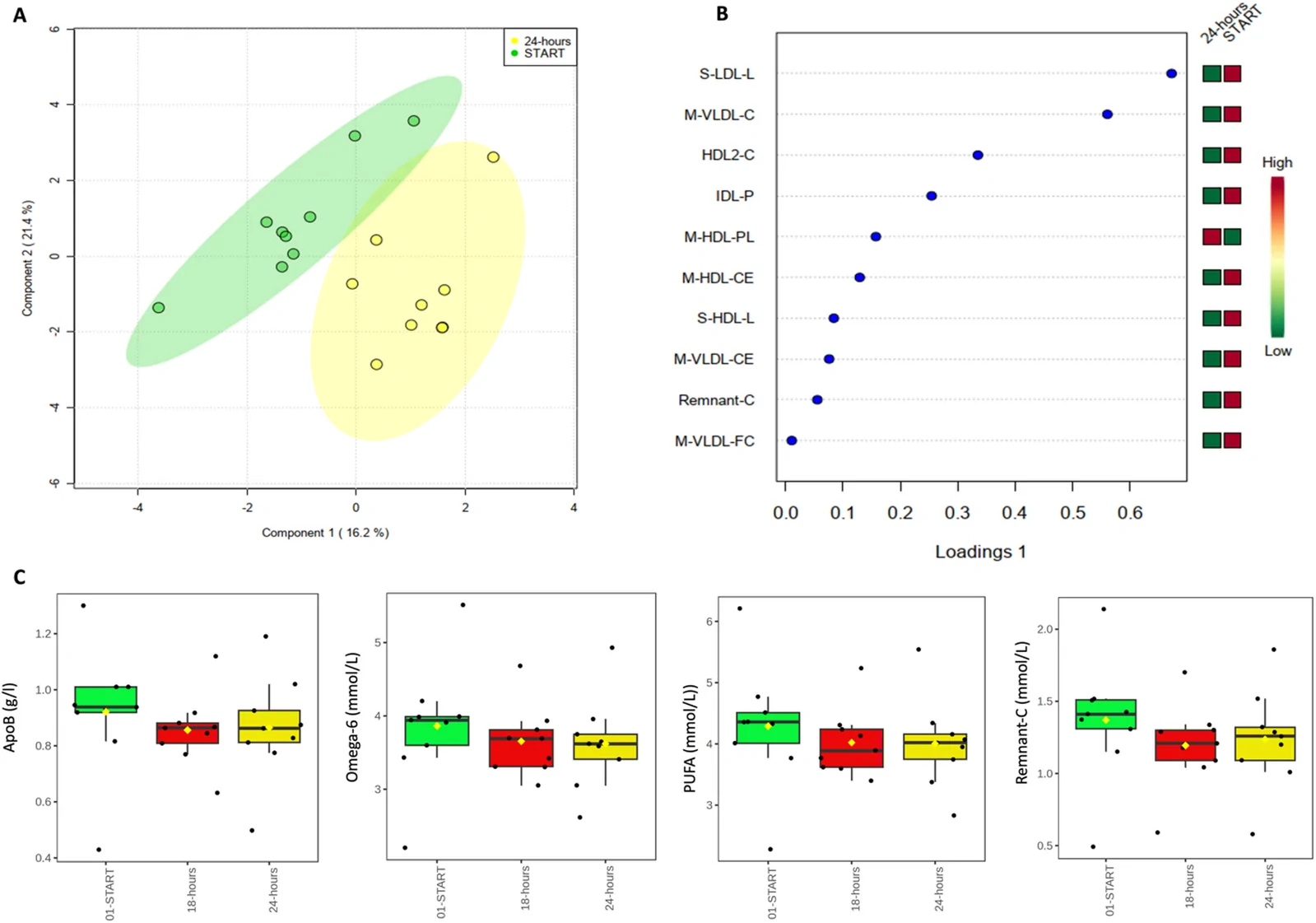
Metabolic response following a short sleep period between gaming sessions. **A** sPLS-DA score plot comparing START (green) and 24 h (yellow; sampled after the short sleep period). **B** Loading plot for the START vs. 24 h comparison. **C** Biochemical changes in four health-related metabolites at START (green), 18 h (red), and 24 h (yellow). In panel C, box plots display the median (horizontal line within the box), interquartile range (box representing the 25th–75th percentiles), and whiskers extending to the most extreme data points within 1.5 × the interquartile range. Individual observations are shown as black dots, and yellow diamonds indicate the mean value at each time point

To assess changes following the sleep period between gaming sessions, serum metabolomic profiles at baseline (START) were compared with those obtained at 24 h using exploratory sPLS-DA. The sPLS-DA score plot showed separation between baseline and 24 h samples (Fig. 4A). The corresponding loading plot illustrates the contribution of individual metabolites to this separation (Fig. 4B). Among selected metabolites, ApoB, Omega-6, PUFA, and remnant cholesterol showed lower values at 24 h compared with baseline, whereas other metabolites showed variable patterns (Fig. 4C).

### Metabolic recovery five days after prolonged gaming

**Fig. 5:**
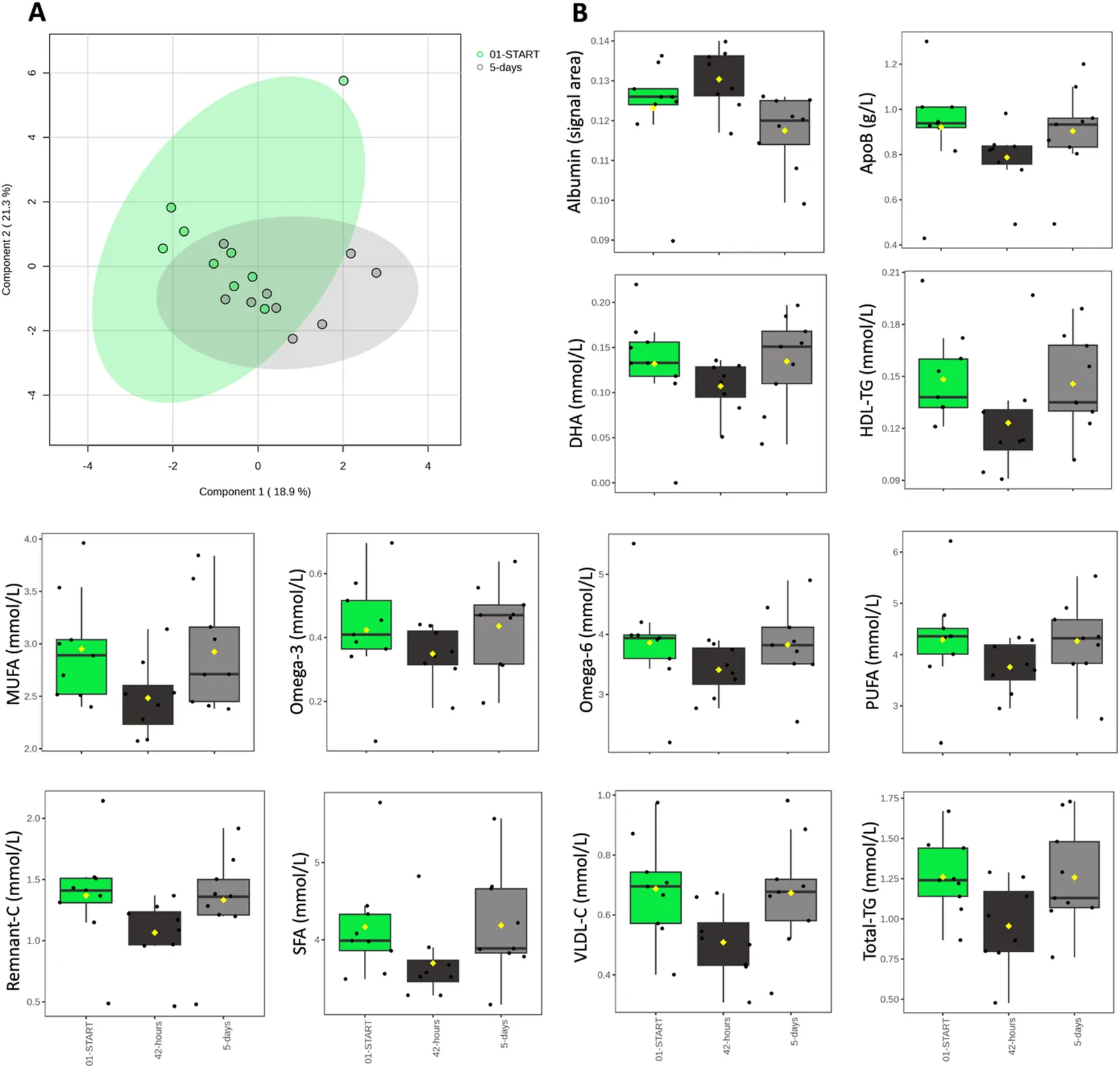
Recovery of metabolic profile after prolonged gaming and 5-day follow-up. **A** sPLS-DA score plot comparing START (green) and 5-day post-gaming (grey). **B** Biochemical changes in the twelve health-related metabolites at START (green), 42 h (dark grey), and 5-day post-gaming. In panel B, box plots display the median (horizontal line within the box), interquartile range (box representing the 25th–75th percentiles), and whiskers extending to the most extreme data points within 1.5 × the interquartile range. Individual observations are shown as black dots, and yellow diamonds indicate the mean value at each time point

To examine recovery following the gaming intervention, serum metabolomic profiles at baseline (START) were compared with profiles obtained five days after completion of the gaming sessions. The sPLS-DA score plot showed substantial overlap between baseline and five-day samples (Fig. 5A), consistent with similarity of the global metabolomic profiles at follow up.

At the individual metabolite level, concentrations at five days were generally closer to baseline values than those observed at the end of the gaming period (Fig. 5B). However, variability between metabolites and between individuals was observed. No consistent directional pattern of sustained metabolomic alteration relative to baseline was identified at the 5-day follow up.

## Discussion

The present study characterized serum metabolomic profiles in healthy young men during prolonged computer gaming conducted under real-world LAN conditions, using dense longitudinal sampling before, during, and after the exposure. Using high-throughput ^1^H-NMR metabolomics, temporal variation was observed across multiple lipid-related measures, particularly within very low-density lipoprotein (VLDL) subclasses, during the gaming period. At five days following the intervention, global metabolomic profiles showed substantial overlap with baseline, indicating convergence toward pre-intervention patterns.

### Metabolomic patterns during prolonged gaming

Across the gaming sessions, temporal variation was most evident in triglyceride-related measures and VLDL subclass variables. The metabolites contributing most strongly to the exploratory multivariate models were predominantly lipid measures within large VLDL subclasses, including total lipid content, cholesterol fractions, and VLDL triglycerides. These observations align with the known sensitivity of triglyceride-rich lipoproteins to short-term metabolic conditions.

The temporal pattern observed across time points suggests gradual changes rather than abrupt shifts in metabolomic profiles. Given the study design, which combined prolonged sitting, irregular sleep, and unrestricted food intake, the observed variation likely reflects the combined influence of multiple behavioral factors. In particular, dietary intake during the gaming sessions, previously reported in this cohort (Kasper B. Krarup et al. 2024a, b; Kasper Bygum Krarup et al., 2022), may have contributed substantially to the observed variation in triglyceride-rich lipoprotein measures. Acute elevations in these metabolites may reflect increased hepatic VLDL secretion together with altered postprandial lipid clearance under conditions of repeated caloric intake, prolonged sitting, and disrupted sleep-wake regulation. Experimental studies have shown that sleep restriction and circadian disruption can influence insulin sensitivity, substrate utilization, and lipid metabolism, potentially contributing to transient changes in circulating VLDL measures (Aho et al., 2016; Russell et al., 2023).

Although metabolomic studies specifically examining prolonged gaming exposure remain limited, previous studies investigating sedentary behavior, physical inactivity, and insufficient sleep have similarly reported alterations in lipid metabolism and triglyceride-rich lipoprotein pathways (Aho et al., 2016; Russell et al., 2023). However, detailed NMR-based characterization of temporal VLDL subclass responses during prolonged gaming scenarios has not previously been reported. Accordingly, the present findings should be interpreted as hypothesis-generating observations reflecting integrated metabolic responses to a complex behavioral exposure rather than the isolated effects of gaming or sedentary activity alone.

VLDL subclasses, particularly larger VLDL particles, have been associated with lipid metabolism and cardiometabolic risk in prior studies (Adiels et al., 2006; Blake et al., 2002; Packard & Shepherd, 1997). In addition, remnant cholesterol and triglyceride-rich lipoproteins have been linked to atherogenic processes (Nordestgaard et al., 2007; Varbo et al., 2013). However, the present study examined an acute exposure in healthy individuals, and the observed metabolomic changes should therefore be interpreted as transient variation over time rather than evidence of sustained dysregulation.

### Metabolomic responses across the sleep interval

The study design allowed comparison of metabolomic profiles before and after the short sleep period between the two gaming sessions. At 24 h, metabolomic profiles differed from baseline, and several lipid-related measures remained altered relative to START. These findings indicate that the metabolic state observed during the first gaming session was not fully aligned with baseline following the sleep interval.

Among selected metabolites, ApoB, Omega-6, PUFA, and remnant cholesterol showed lower values at 24 h compared with baseline. In addition, reduced values of total lipids in small HDL (S-HDL-L) were observed at 18 h and remained lower at 24 h. Small HDL particles are involved in lipid exchange processes, including interactions with triglyceride-rich lipoproteins (Kontush & John Chapman, 2006; Rye & Barter, 2014; Tall, 1990). However, the present study was not designed to assess functional aspects of HDL metabolism, and these observations should be interpreted as compositional changes in HDL subclasses.

Albumin concentrations increased toward the end of the intervention. While this pattern may reflect changes in hydration status during prolonged gaming, the present data do not allow direct assessment of underlying mechanisms.

### Recovery of metabolomic profiles following gaming

At five days following the intervention, metabolomic profiles showed substantial overlap with baseline in multivariate space, and most metabolites that varied during the gaming period returned toward baseline levels. These findings indicate that, at the group level, the metabolomic profile following a single prolonged gaming exposure converged toward pre-intervention patterns.

Despite this overall convergence, variability between individuals was observed at follow-up for several metabolites. Such variability may reflect differences in post-intervention behaviors, including dietary intake, sleep patterns, and physical activity, which were not controlled in the recovery period.

The absence of consistent directional differences at five days suggests that the metabolic alterations observed during the gaming sessions were largely transient in this cohort. However, the present findings do not address the effects of repeated or habitual exposure to similar behavioral conditions. Future studies are needed to examine whether recurrent prolonged gaming, in combination with sedentary behavior, sleep disruption, and dietary patterns, may contribute to cumulative changes in lipid-related metabolomic profiles over time. Accordingly, the present findings should be interpreted as hypothesis-generating observations requiring confirmation in larger controlled studies.

## Strengths, limitations, and implications for future work

A major strength of the present study is the dense longitudinal sampling across a prolonged real-world gaming scenario combined with high-throughput ^1^H-NMR metabolomics to quantify detailed lipoprotein subclass measures. The repeated within-subject design enabled characterisation of temporal variation during both the gaming exposure and short-term recovery. The Nightingale NMR platform provides broad coverage of lipoprotein subclasses, lipid fractions, fatty acids, and related metabolites, allowing assessment of lipid-related changes beyond conventional clinical lipid panels. In the same cohort, routine clinical lipid measurements did not detect clear temporal variation in total cholesterol or triglycerides (Kasper B. Krarup et al. 2024a, b), whereas the NMR platform identified variation across VLDL subclasses, illustrating the sensitivity of high-resolution lipoprotein profiling.

Several limitations should be considered. First, the sample size was small, and the study was designed as an intensive repeated-measures investigation rather than a population-based analysis. Accordingly, the findings should be interpreted within this exploratory framework, and generalizability to other populations is limited.

Second, the exposure represented a composite behavioral scenario incorporating prolonged sitting, sleep restriction, circadian disruption, emotional engagement, and unrestricted food and beverage intake. These factors were not experimentally separated; therefore, their independent contributions to the observed metabolomic changes cannot be determined.

The absence of a non-gaming control condition, controlled feeding condition, and sleep-controlled comparison group further limits causal attribution. Therefore, the findings should be interpreted as the integrated metabolomic response to a real-world prolonged gaming scenario rather than as the isolated effect of gaming or sedentary behavior alone.

Dietary intake during the gaming sessions was recorded and reported in a previous publication from the same cohort (Kasper Bygum Krarup et al., 2022). However, in the present metabolomic analysis, dietary intake was not incorporated quantitatively into the statistical models. Given the well-established postprandial responsiveness of triglycerides and VLDL subclasses, the observed variation in triglyceride-rich lipoprotein measures likely reflects the combined influence of dietary intake and sedentary exposure, and the independent contributions of these factors could not be evaluated within the present study design.

Although follow-up sampling at five days indicated convergence of metabolomic profiles toward baseline, longer-term trajectories and the effects of repeated exposure were not assessed. Exploratory regression analyses between metabolite changes and physiological parameters (e.g., heart rate and blood pressure) were considered; however, the limited sample size in this pilot study restricted statistical power for robust multivariable association analyses. This will be an important focus of future studies with larger cohorts.

The present findings provide time-resolved insight into metabolomic variation during prolonged gaming exposure. Future studies should extend these observations to real-world populations with habitual high gaming exposure and incorporate detailed assessment of dietary intake, sleep, and physical activity. Such approaches are needed to clarify the relative contributions of behavioral factors and to determine whether repeated exposures are associated with cumulative metabolic changes over time.

## Conclusions

This exploratory study shows that prolonged computer gaming sessions are associated with transient alterations in serum metabolomic profiles in healthy young men, with the most prominent changes observed in lipid-related measures, particularly within very low-density lipoprotein subclasses.

At five days following the intervention, metabolomic profiles showed substantial overlap with baseline, and most metabolites that varied during the gaming period returned toward baseline concentrations, suggesting convergence of the global metabolomic profile after a single gaming episode.

No consistent evidence of sustained global metabolic alteration was observed at five days. However, the present findings do not address the effects of repeated exposure. Further studies incorporating larger cohorts, repeated gaming scenarios, and detailed behavioral assessment are needed to determine whether recurrent exposure to prolonged gaming conditions may be associated with cumulative changes in lipid-related metabolomic profiles.

## Supplementary Information

Below is the link to the electronic supplementary material.

Supplementary material 1 (DOCX 44.8 kb)

## Data Availability

The datasets generated during and/or analyzed during the current study are available from the corresponding author upon reasonable request.
