# Supplementary material for: Exploratory NMR-based metabolomics reveals transient lipoprotein changes during prolonged computer gaming
Source: Metabolomics. 2026 Jul 3;22(4):117. doi: 10.1007/s11306-026-02502-9 (PMC13331907; doi:10.1007/s11306-026-02502-9)
Supplement: Supplementary file 1 — Supplementary material 1 (DOCX 44.8 kb) [file 11306_2026_2502_MOESM1_ESM.docx]

**Table S1.** Internal 5-fold cross-validation error rates for pairwise sPLS-DA models

| **sPLS-DA comparison** | **Comp.1 error (%)** | **Comp. 2 error (%)** | **Comp.3 error (%)** | **Comp.4 error (%)** | **Comp. 5 error (%)** | **Lowest error (%)** | **Best component** |
| --- | --- | --- | --- | --- | --- | --- | --- |
| START vs 6 h | 27.8 | 44.4 | 55.6 | 44.4 | 38.9 | 27.8 | 1 |
| START vs 12 h | 55.6 | 50.0 | 38.9 | 44.4 | 33.3 | 33.3 | 5 |
| START vs 18 h | 50.0 | 44.4 | 55.6 | 50.0 | 27.8 | 27.8 | 5 |
| START vs 30 h | 16.7 | 16.7 | 27.8 | 33.3 | 38.9 | 16.7 | 1–2 |
| START vs 36 h | 44.4 | 50.0 | 38.9 | 33.3 | 44.4 | 33.3 | 4 |
| START vs 42 h | 41.2 | 41.2 | 29.4 | 35.3 | 29.4 | 29.4 | 3 and 5 |

Error rates were obtained from internal 5-fold cross-validation of pairwise sPLS-DA models. Lower values indicate better classification performance. Results were used to assess internal model stability and should be interpreted cautiously because of the exploratory design, small sample size, and repeated-measures structure.

**Table S2** List of 128 metabolites identified by serum metabolomics

| **No** | **Metabolites** | **Short name** | **Group** |
| --- | --- | --- | --- |
| 1 | Total cholesterol | Total-C | Cholesterol |
| 2 | VLDL cholesterol | VLDL-C | Cholesterol |
| 3 | Remnant cholesterol (non-HDL, non-LDL -cholesterol) | Remnant-C | Cholesterol |
| 4 | LDL cholesterol | LDL-C | Cholesterol |
| 5 | HDL cholesterol | HDL-C | Cholesterol |
| 6 | HDL2 cholesterol | HDL2-C | Cholesterol |
| 7 | HDL3 cholesterol | HDL3-C | Cholesterol |
| 8 | Total esterified cholesterol | Esterified-C | Cholesterol |
| 9 | Total free cholesterol | Free-C | Cholesterol |
| 10 | Total triglycerides | Total-TG | Glycerides and phospholipids |
| 11 | Triglycerides in VLDL | VLDL-TG | Glycerides and phospholipids |
| 12 | Triglycerides in LDL | LDL-TG | Glycerides and phospholipids |
| 13 | Triglycerides in HDL | HDL-TG | Glycerides and phospholipids |
| 14 | Phosphoglycerides | Phosphoglyc | Glycerides and phospholipids |
| 15 | Total cholines | Cholines | Glycerides and phospholipids |
| 16 | Phosphatidylcholines | Phosphatidylc | Glycerides and phospholipids |
| 17 | Sphingomyelins | Sphingomyelins | Glycerides and phospholipids |
| 18 | Apolipoprotein B | ApoB | Apolipoproteins |
| 19 | Apolipoprotein A1 | ApoA1 | Apolipoproteins |
| 20 | Total fatty acids | Total-FA | Fatty acids |
| 21 | Degree of unsaturation | Unsaturation | Fatty acids |
| 22 | Omega-3 fatty acids | Omega-3 | Fatty acids |
| 23 | Omega-6 fatty acids | Omega-6 | Fatty acids |
| 24 | Polyunsaturated fatty acids | PUFA | Fatty acids |
| 25 | Monounsaturated fatty acids | MUFA | Fatty acids |
| 26 | Saturated fatty acids | SFA | Fatty acids |
| 27 | Linoleic acid | LA | Fatty acids |
| 28 | Docosahexaenoic acid | DHA | Fatty acids |
| 29 | Albumin | Albumin | Fluid balance |
| 30 | Glycoprotein acetyls | GlycA | Inflammation |
| 31 | Concentration of chylomicrons and extremely large VLDL particles | XXL-VLDL-P | Lipoprotein subclasses |
| 32 | Total lipids in chylomicrons and extremely large VLDL | XXL-VLDL-L | Lipoprotein subclasses |
| 33 | Phospholipids in chylomicrons and extremely large VLDL | XXL-VLDL-PL | Lipoprotein subclasses |
| 34 | Cholesterol in chylomicrons and extremely large VLDL | XXL-VLDL-C | Lipoprotein subclasses |
| 35 | Cholesteryl esters in chylomicrons and extremely large VLDL | XXL-VLDL-CE | Lipoprotein subclasses |
| 36 | Free cholesterol in chylomicrons and extremely large VLDL | XXL-VLDL-FC | Lipoprotein subclasses |
| 37 | Triglycerides in chylomicrons and extremely large VLDL | XXL-VLDL-TG | Lipoprotein subclasses |
| 38 | Concentration of very large VLDL particles | XL-VLDL-P | Lipoprotein subclasses |
| 39 | Total lipids in very large VLDL | XL-VLDL-L | Lipoprotein subclasses |
| 40 | Phospholipids in very large VLDL | XL-VLDL-PL | Lipoprotein subclasses |
| 41 | Cholesterol in very large VLDL | XL-VLDL-C | Lipoprotein subclasses |
| 42 | Cholesteryl esters in very large VLDL | XL-VLDL-CE | Lipoprotein subclasses |
| 43 | Free cholesterol in very large VLDL | XL-VLDL-FC | Lipoprotein subclasses |
| 44 | Triglycerides in very large VLDL | XL-VLDL-TG | Lipoprotein subclasses |
| 45 | Concentration of large VLDL particles | L-VLDL-P | Lipoprotein subclasses |
| 46 | Total lipids in large VLDL | L-VLDL-L | Lipoprotein subclasses |
| 47 | Phospholipids in large VLDL | L-VLDL-PL | Lipoprotein subclasses |
| 48 | Cholesterol in large VLDL | L-VLDL-C | Lipoprotein subclasses |
| 49 | Cholesteryl esters in large VLDL | L-VLDL-CE | Lipoprotein subclasses |
| 50 | Free cholesterol in large VLDL | L-VLDL-FC | Lipoprotein subclasses |
| 51 | Triglycerides in large VLDL | L-VLDL-TG | Lipoprotein subclasses |
| 52 | Concentration of medium VLDL particles | M-VLDL-P | Lipoprotein subclasses |
| 53 | Total lipids in medium VLDL | M-VLDL-L | Lipoprotein subclasses |
| 54 | Phospholipids in medium VLDL | M-VLDL-PL | Lipoprotein subclasses |
| 55 | Cholesterol in medium VLDL | M-VLDL-C | Lipoprotein subclasses |
| 56 | Cholesteryl esters in medium VLDL | M-VLDL-CE | Lipoprotein subclasses |
| 57 | Free cholesterol in medium VLDL | M-VLDL-FC | Lipoprotein subclasses |
| 58 | Triglycerides in medium VLDL | M-VLDL-TG | Lipoprotein subclasses |
| 59 | Concentration of small VLDL particles | S-VLDL-P | Lipoprotein subclasses |
| 60 | Total lipids in small VLDL | S-VLDL-L | Lipoprotein subclasses |
| 61 | Phospholipids in small VLDL | S-VLDL-PL | Lipoprotein subclasses |
| 62 | Cholesterol in small VLDL | S-VLDL-C | Lipoprotein subclasses |
| 63 | Cholesteryl esters in small VLDL | S-VLDL-CE | Lipoprotein subclasses |
| 64 | Free cholesterol in small VLDL | S-VLDL-FC | Lipoprotein subclasses |
| 65 | Triglycerides in small VLDL | S-VLDL-TG | Lipoprotein subclasses |
| 66 | Concentration of very small VLDL particles | XS-VLDL-P | Lipoprotein subclasses |
| 67 | Total lipids in very small VLDL | XS-VLDL-L | Lipoprotein subclasses |
| 68 | Phospholipids in very small VLDL | XS-VLDL-PL | Lipoprotein subclasses |
| 69 | Cholesterol in very small VLDL | XS-VLDL-C | Lipoprotein subclasses |
| 70 | Cholesteryl esters in very small VLDL | XS-VLDL-CE | Lipoprotein subclasses |
| 71 | Free cholesterol in very small VLDL | XS-VLDL-FC | Lipoprotein subclasses |
| 72 | Triglycerides in very small VLDL | XS-VLDL-TG | Lipoprotein subclasses |
| 73 | Concentration of IDL particles | IDL-P | Lipoprotein subclasses |
| 74 | Total lipids in IDL | IDL-L | Lipoprotein subclasses |
| 75 | Phospholipids in IDL | IDL-PL | Lipoprotein subclasses |
| 76 | Cholesterol in IDL | IDL-C | Lipoprotein subclasses |
| 77 | Cholesteryl esters in IDL | IDL-CE | Lipoprotein subclasses |
| 78 | Free cholesterol in IDL | IDL-FC | Lipoprotein subclasses |
| 79 | Triglycerides in IDL | IDL-TG | Lipoprotein subclasses |
| 80 | Concentration of large LDL particles | L-LDL-P | Lipoprotein subclasses |
| 81 | Total lipids in large LDL | L-LDL-L | Lipoprotein subclasses |
| 82 | Phospholipids in large LDL | L-LDL-PL | Lipoprotein subclasses |
| 83 | Cholesterol in large LDL | L-LDL-C | Lipoprotein subclasses |
| 84 | Cholesteryl esters in large LDL | L-LDL-CE | Lipoprotein subclasses |
| 85 | Free cholesterol in large LDL | L-LDL-FC | Lipoprotein subclasses |
| 86 | Triglycerides in large LDL | L-LDL-TG | Lipoprotein subclasses |
| 87 | Concentration of medium LDL particles | M-LDL-P | Lipoprotein subclasses |
| 88 | Total lipids in medium LDL | M-LDL-L | Lipoprotein subclasses |
| 89 | Phospholipids in medium LDL | M-LDL-PL | Lipoprotein subclasses |
| 90 | Cholesterol in medium LDL | M-LDL-C | Lipoprotein subclasses |
| 91 | Cholesteryl esters in medium LDL | M-LDL-CE | Lipoprotein subclasses |
| 92 | Free cholesterol in medium LDL | M-LDL-FC | Lipoprotein subclasses |
| 93 | Triglycerides in medium LDL | M-LDL-TG | Lipoprotein subclasses |
| 94 | Concentration of small LDL particles | S-LDL-P | Lipoprotein subclasses |
| 95 | Total lipids in small LDL | S-LDL-L | Lipoprotein subclasses |
| 96 | Phospholipids in small LDL | S-LDL-PL | Lipoprotein subclasses |
| 97 | Cholesterol in small LDL | S-LDL-C | Lipoprotein subclasses |
| 98 | Cholesteryl esters in small LDL | S-LDL-CE | Lipoprotein subclasses |
| 99 | Free cholesterol in small LDL | S-LDL-FC | Lipoprotein subclasses |
| 100 | Triglycerides in small LDL | S-LDL-TG | Lipoprotein subclasses |
| 101 | Concentration of very large HDL particles | XL-HDL-P | Lipoprotein subclasses |
| 102 | Total lipids in very large HDL | XL-HDL-L | Lipoprotein subclasses |
| 103 | Phospholipids in very large HDL | XL-HDL-PL | Lipoprotein subclasses |
| 104 | Cholesterol in very large HDL | XL-HDL-C | Lipoprotein subclasses |
| 105 | Cholesteryl esters in very large HDL | XL-HDL-CE | Lipoprotein subclasses |
| 106 | Free cholesterol in very large HDL | XL-HDL-FC | Lipoprotein subclasses |
| 107 | Triglycerides in very large HDL | XL-HDL-TG | Lipoprotein subclasses |
| 108 | Concentration of large HDL particles | L-HDL-P | Lipoprotein subclasses |
| 109 | Total lipids in large HDL | L-HDL-L | Lipoprotein subclasses |
| 110 | Phospholipids in large HDL | L-HDL-PL | Lipoprotein subclasses |
| 111 | Cholesterol in large HDL | L-HDL-C | Lipoprotein subclasses |
| 112 | Cholesteryl esters in large HDL | L-HDL-CE | Lipoprotein subclasses |
| 113 | Free cholesterol in large HDL | L-HDL-FC | Lipoprotein subclasses |
| 114 | Triglycerides in large HDL | L-HDL-TG | Lipoprotein subclasses |
| 115 | Concentration of medium HDL particles | M-HDL-P | Lipoprotein subclasses |
| 116 | Total lipids in medium HDL | M-HDL-L | Lipoprotein subclasses |
| 117 | Phospholipids in medium HDL | M-HDL-PL | Lipoprotein subclasses |
| 118 | Cholesterol in medium HDL | M-HDL-C | Lipoprotein subclasses |
| 119 | Cholesteryl esters in medium HDL | M-HDL-CE | Lipoprotein subclasses |
| 120 | Free cholesterol in medium HDL | M-HDL-FC | Lipoprotein subclasses |
| 121 | Triglycerides in medium HDL | M-HDL-TG | Lipoprotein subclasses |
| 122 | Concentration of small HDL particles | S-HDL-P | Lipoprotein subclasses |
| 123 | Total lipids in small HDL | S-HDL-L | Lipoprotein subclasses |
| 124 | Phospholipids in small HDL | S-HDL-PL | Lipoprotein subclasses |
| 125 | Cholesterol in small HDL | S-HDL-C | Lipoprotein subclasses |
| 126 | Cholesteryl esters in small HDL | S-HDL-CE | Lipoprotein subclasses |
| 127 | Free cholesterol in small HDL | S-HDL-FC | Lipoprotein subclasses |
| 128 | Triglycerides in small HDL | S-HDL-TG | Lipoprotein subclasses |

**Table S3.** ANOVA result for all 128 metabolites identified by serum metabolomics

| **Metabolites** | **F.stat** | **p.value** | **-log10(p)** | **FDR** |
| --- | --- | --- | --- | --- |
| XXL-VLDL-CE | 6,6825 | 6,38E-06 | 5,1951 | 0,00055164 |
| XXL-VLDL-C | 6,5212 | 8,55E-06 | 5,0679 | 0,00055164 |
| XL-VLDL-CE | 5,9812 | 2,32E-05 | 4,6346 | 0,00099736 |
| XL-VLDL-C | 5,8088 | 3,21E-05 | 4,4939 | 0,0010342 |
| XL-VLDL-FC | 5,4937 | 5,84E-05 | 4,2339 | 0,0015056 |
| XXL-VLDL-L | 5,2392 | 9,52E-05 | 4,0211 | 0,0017314 |
| XXL-VLDL-P | 5,1861 | 0,00010558 | 3,9764 | 0,0017314 |
| XL-VLDL-PL | 5,0899 | 0,0001273 | 3,8952 | 0,0017314 |
| XXL-VLDL-FC | 5,0892 | 0,00012748 | 3,8946 | 0,0017314 |
| XXL-VLDL-PL | 5,0135 | 0,00014776 | 3,8304 | 0,0017314 |
| XL-VLDL-L | 4,9724 | 0,00016014 | 3,7955 | 0,0017314 |
| XXL-VLDL-TG | 4,9527 | 0,00016646 | 3,7787 | 0,0017314 |
| XL-VLDL-P | 4,8972 | 0,00018561 | 3,7314 | 0,0017314 |
| L-VLDL-C | 4,891 | 0,0001879 | 3,7261 | 0,0017314 |
| L-VLDL-FC | 4,8334 | 0,00021044 | 3,6769 | 0,0018098 |
| Total-TG | 4,648 | 0,00030366 | 3,5176 | 0,0023069 |
| XL-VLDL-TG | 4,6475 | 0,000304 | 3,5171 | 0,0023069 |
| L-VLDL-CE | 4,5752 | 0,00035103 | 3,4547 | 0,0025157 |
| L-VLDL-L | 4,497 | 0,00041024 | 3,387 | 0,0026644 |
| L-VLDL-PL | 4,4834 | 0,00042156 | 3,3751 | 0,0026644 |
| MUFA | 4,4683 | 0,00043449 | 3,362 | 0,0026644 |
| L-VLDL-P | 4,4434 | 0,0004567 | 3,3404 | 0,0026644 |
| M-VLDL-FC | 4,4237 | 0,00047505 | 3,3233 | 0,0026644 |
| L-VLDL-TG | 4,3115 | 0,00059507 | 3,2254 | 0,0031985 |
| VLDL-TG | 4,2706 | 0,00064617 | 3,1897 | 0,0033343 |
| M-VLDL-L | 4,2201 | 0,00071556 | 3,1454 | 0,0035503 |
| M-VLDL-P | 4,1711 | 0,00078995 | 3,1024 | 0,0037742 |
| M-VLDL-PL | 4,0321 | 0,0010472 | 2,98 | 0,0048246 |
| M-VLDL-TG | 3,9709 | 0,001186 | 2,9259 | 0,0052756 |
| M-VLDL-C | 3,8577 | 0,0014939 | 2,8257 | 0,006424 |
| XL-HDL-TG | 3,7585 | 0,0018302 | 2,7375 | 0,0076158 |
| S-VLDL-TG | 3,3095 | 0,0046178 | 2,3356 | 0,018051 |
| HDL-TG | 2,8937 | 0,010948 | 1,9607 | 0,041539 |
| M-VLDL-CE | 2,713 | 0,01594 | 1,7975 | 0,05875 |
| SFA | 2,6127 | 0,019628 | 1,7071 | 0,070335 |
| S-VLDL-P | 2,4786 | 0,025912 | 1,5865 | 0,090341 |
| XS-VLDL-TG | 2,3963 | 0,030714 | 1,5127 | 0,10426 |
| Total-FA | 2,3244 | 0,035619 | 1,4483 | 0,11782 |
| S-VLDL-L | 2,2629 | 0,040413 | 1,3935 | 0,13033 |
| S-HDL-CE | 2,2127 | 0,044791 | 1,3488 | 0,14093 |
| VLDL-C | 2,1687 | 0,049004 | 1,3098 | 0,15051 |
| S-VLDL-PL | 2,0648 | 0,060539 | 1,218 | 0,18162 |
| S-VLDL-FC | 1,9703 | 0,073258 | 1,1351 | 0,21478 |
| S-HDL-C | 1,9521 | 0,075994 | 1,1192 | 0,21785 |
| S-HDL-TG | 1,848 | 0,093546 | 1,029 | 0,26234 |
| M-HDL-TG | 1,6821 | 0,12964 | 0,88725 | 0,35583 |
| S-HDL-FC | 1,2661 | 0,28157 | 0,55042 | 0,75672 |
| GlycA | 1,213 | 0,309 | 0,51004 | 0,8135 |
| S-LDL-TG | 1,126 | 0,35837 | 0,44567 | 0,92459 |
| S-HDL-PL | 1,0979 | 0,37557 | 0,42531 | 0,94997 |
| Phosphoglyc | 0,99827 | 0,44101 | 0,35555 | 0,99288 |
| LA | 0,95285 | 0,47315 | 0,325 | 0,99288 |
| L-HDL-TG | 0,9509 | 0,47457 | 0,3237 | 0,99288 |
| Phosphatidylc | 0,93019 | 0,4897 | 0,31007 | 0,99288 |
| S-VLDL-C | 0,89961 | 0,51252 | 0,29029 | 0,99288 |
| LDL-TG | 0,82442 | 0,5708 | 0,24351 | 0,99288 |
| Remnant-C | 0,82154 | 0,57309 | 0,24178 | 0,99288 |
| ApoB | 0,80974 | 0,58249 | 0,23472 | 0,99288 |
| Albumin | 0,80691 | 0,58475 | 0,23303 | 0,99288 |
| Cholines | 0,78348 | 0,60359 | 0,21926 | 0,99288 |
| S-HDL-L | 0,77557 | 0,61 | 0,21467 | 0,99288 |
| M-HDL-CE | 0,76963 | 0,61481 | 0,21126 | 0,99288 |
| M-HDL-C | 0,7403 | 0,63873 | 0,19468 | 0,99288 |
| XS-VLDL-P | 0,73781 | 0,64077 | 0,1933 | 0,99288 |
| Omega-6 | 0,72778 | 0,649 | 0,18776 | 0,99288 |
| S-HDL-P | 0,72731 | 0,64938 | 0,1875 | 0,99288 |
| PUFA | 0,70471 | 0,66796 | 0,17525 | 0,99288 |
| IDL-TG | 0,69485 | 0,67607 | 0,17001 | 0,99288 |
| XS-VLDL-L | 0,66109 | 0,70384 | 0,15253 | 0,99288 |
| XL-HDL-CE | 0,65979 | 0,70491 | 0,15187 | 0,99288 |
| S-VLDL-CE | 0,65143 | 0,71176 | 0,14767 | 0,99288 |
| HDL3-C | 0,63208 | 0,72758 | 0,13812 | 0,99288 |
| M-HDL-L | 0,63063 | 0,72876 | 0,13741 | 0,99288 |
| M-HDL-P | 0,62555 | 0,7329 | 0,13495 | 0,99288 |
| M-HDL-PL | 0,62258 | 0,73532 | 0,13352 | 0,99288 |
| Omega-3 | 0,60374 | 0,75057 | 0,12461 | 0,99288 |
| XS-VLDL-CE | 0,59731 | 0,75574 | 0,12163 | 0,99288 |
| M-HDL-FC | 0,5903 | 0,76136 | 0,11841 | 0,99288 |
| XS-VLDL-PL | 0,57899 | 0,77038 | 0,1133 | 0,99288 |
| XL-HDL-C | 0,55485 | 0,78939 | 0,10271 | 0,99288 |
| S-LDL-CE | 0,53451 | 0,80513 | 0,094136 | 0,99288 |
| XS-VLDL-C | 0,52153 | 0,815 | 0,088844 | 0,99288 |
| M-LDL-CE | 0,519 | 0,8169 | 0,087828 | 0,99288 |
| ApoA1 | 0,48519 | 0,84184 | 0,07477 | 0,99288 |
| M-LDL-TG | 0,47399 | 0,84984 | 0,070661 | 0,99288 |
| XS-VLDL-FC | 0,4645 | 0,85652 | 0,067264 | 0,99288 |
| DHA | 0,43534 | 0,87634 | 0,057327 | 0,99288 |
| Unsaturation | 0,42129 | 0,88549 | 0,052815 | 0,99288 |
| IDL-FC | 0,40778 | 0,89403 | 0,048649 | 0,99288 |
| M-LDL-C | 0,4068 | 0,89463 | 0,048355 | 0,99288 |
| LDL-C | 0,40226 | 0,89743 | 0,046997 | 0,99288 |
| XL-HDL-FC | 0,39787 | 0,90012 | 0,045701 | 0,99288 |
| S-LDL-C | 0,39336 | 0,90284 | 0,044391 | 0,99288 |
| IDL-P | 0,38469 | 0,90797 | 0,041928 | 0,99288 |
| Sphingomyelins | 0,38291 | 0,90901 | 0,041431 | 0,99288 |
| L-LDL-TG | 0,37625 | 0,91286 | 0,039596 | 0,99288 |
| IDL-L | 0,37415 | 0,91406 | 0,039027 | 0,99288 |
| XL-HDL-P | 0,36992 | 0,91645 | 0,037893 | 0,99288 |
| XL-HDL-L | 0,36879 | 0,91708 | 0,037593 | 0,99288 |
| L-LDL-FC | 0,35643 | 0,92386 | 0,034395 | 0,99288 |
| L-LDL-CE | 0,35189 | 0,92628 | 0,033255 | 0,99288 |
| L-LDL-C | 0,34587 | 0,92944 | 0,031778 | 0,99288 |
| IDL-PL | 0,3264 | 0,9392 | 0,027243 | 0,99288 |
| M-LDL-P | 0,31066 | 0,94657 | 0,023849 | 0,99288 |
| M-LDL-L | 0,31026 | 0,94675 | 0,023765 | 0,99288 |
| L-LDL-L | 0,30973 | 0,94699 | 0,023657 | 0,99288 |
| IDL-C | 0,30967 | 0,94701 | 0,023643 | 0,99288 |
| Free-C | 0,30522 | 0,949 | 0,022734 | 0,99288 |
| L-LDL-P | 0,30136 | 0,95069 | 0,02196 | 0,99288 |
| IDL-CE | 0,2959 | 0,95304 | 0,020889 | 0,99288 |
| L-LDL-PL | 0,28033 | 0,9594 | 0,018001 | 0,99288 |
| Total-C | 0,26619 | 0,96475 | 0,015586 | 0,99288 |
| S-LDL-L | 0,25788 | 0,9677 | 0,014258 | 0,99288 |
| S-LDL-P | 0,25684 | 0,96806 | 0,014098 | 0,99288 |
| Esterified-C | 0,25377 | 0,96911 | 0,013626 | 0,99288 |
| L-HDL-CE | 0,2354 | 0,97498 | 0,011004 | 0,99288 |
| XL-HDL-PL | 0,23398 | 0,97541 | 0,010814 | 0,99288 |
| HDL-C | 0,22885 | 0,97691 | 0,010146 | 0,99288 |
| L-HDL-P | 0,22196 | 0,97884 | 0,0092883 | 0,99288 |
| M-LDL-PL | 0,22094 | 0,97912 | 0,0091653 | 0,99288 |
| L-HDL-L | 0,21451 | 0,98082 | 0,0084114 | 0,99288 |
| M-LDL-FC | 0,21423 | 0,98089 | 0,0083793 | 0,99288 |
| L-HDL-C | 0,21213 | 0,98143 | 0,0081421 | 0,99288 |
| L-HDL-PL | 0,1982 | 0,98476 | 0,0066701 | 0,99288 |
| S-LDL-PL | 0,19814 | 0,98477 | 0,0066642 | 0,99288 |
| S-LDL-FC | 0,1824 | 0,98807 | 0,0052108 | 0,99288 |
| HDL2-C | 0,17752 | 0,989 | 0,0048036 | 0,99288 |
| L-HDL-FC | 0,15368 | 0,99288 | 0,0031016 | 0,99288 |
